# Supplementary material for: Metabolic Adaptations Determine the Evolutionary Trajectory of TOR Signaling in Diverse Eukaryotes
Source: Biomolecules. 2025 Sep 8;15(9):1295. doi: 10.3390/biom15091295 (PMC12467227; doi:10.3390/biom15091295)
Supplement: Supplementary file 1 [file biomolecules-15-01295-s001.zip › biomolecules-3842147-supplemental-figures-title.pdf]

biomolecules

MDPI

## Supplemental Figure Legends

**Figure S1.** Example graphs used to establish HMMER score thresholds and assess protein presence. **(A)** Representative plots used to define scoring thresholds for HMMER alignments. Each graph plots overall bit score (X-axis) against best single domain score (Y-axis) for selected hits across the full range of values. Randomly sampled points were validated via BLAST to assess accuracy. This procedure was applied to all proteins included in the analysis—RICTOR, RAPTOR, TOR, LST8, and SIN1—within all clades. BLAST results confirmed that hits with bit scores <100 were false positives and were excluded. A cutoff region (shaded in purple) was drawn to indicate this threshold. In addition, hits were discarded if either the overall or best single domain score fell below 100 (indicated by red-shaded zones). Scores between 100 and 150 were classified as "low-confidence" based on validation outcomes (white region I); scores between 150 and 300 as "medium-confidence" (white region II); and scores >300 as "high-confidence" detections. **(B)** Box plots showing overall HMMER scores for each TOR complex protein, grouped by clade and BUSCO completeness. These plots illustrate the presence and relative alignment strength of RICTOR, RAPTOR, TOR, LST8, and SIN1 among major eukaryotic clades. For example, RICTOR was detected in Metamonada, Discoba, Alveolata, Rhizaria, and Stramenopiles, but absent from all Archaeplastida clades. A final box plot shows BUSCO completeness scores for all organisms by clade, used to assess assembly quality and determine whether missing components are likely to reflect true absence.

**Figure S2**. Example multiple sequence alignments (MSAs). **(A)** Representative sections of the MSA for RICTOR sequences from Stramenopiles. Expanded alignments show the N-terminal (RICTORN) and middle (RICTORM) domains, respectively. Reference sequences from *H. sapiens*, *S. cerevisiae*, and *S. pombe* are displayed at the top. Position numbering corresponds to the *H. sapiens* RICTOR sequence. Below the references, Stramenopile sequences are ordered by detection confidence based on HMMER scores (high, medium, low). Alignment quality and consensus scores are shown below each alignment window. **(B)** Representative sections of the MSA for RAPTOR sequences from Stramenopiles. Expanded views show alignments at the RAPTOR N-terminus (RAPTORN) and armadillo-repeat (ARM) domains, respectively. As in panel A, reference sequences appear at the top, followed by Stramenopile sequences ordered by HMMER score. No low-confidence RAPTOR hits were observed, so only high- and mediumconfidence sequences are shown. Alignment quality and consensus are displayed below each alignment window.

**Figure S3.** Phylogenetic tree of the Excavates (Discoba and Metamonada). Species labels are color-coded by BUSCO completeness scores (C scores) to indicate overall genome quality. The first heatmap shows HMMER-based detection scores for core TOR complex components: SIN1, RICTOR, RAPTOR, LST8, and TOR. The second heatmap indicates inferred metabolic strategies, as determined by literature review and phylogenetic context. All Discoba and Metamonada species were found to contain both RICTOR and RAPTOR, consistent with the presence of both TORC1 and TORC2. Although SIN1 was detected in only two species, its presence within the Metamonada suggests it may have been present in the Last Eukaryotic Common Ancestor (LECA).

**Figure S4.** Expanded phylogenetic tree of Chlorophyta, Rhodophyta, and Streptophyta. **(A)** Phylogenetic tree of Chlorophyta and Rhodophyta. Species labels are color-

coded by BUSCO completeness score. The first heatmap displays HMMER scores for TOR complex components: SIN1, RICTOR, RAPTOR, LST8, and TOR. The second heatmap shows inferred metabolic strategies based on literature review and phylogenetic context. No TORC2 components were detected in any Chlorophyta or Rhodophyta species, including those classified as mixotrophic or heterotrophic. **(B)** Phylogenetic tree of Streptophyta. For formatting purposes, species labels are omitted; instead, terminal branches are color-coded by BUSCO completeness score. A full list of species is available in Table S1. Consistent with panel A, no RICTOR or SIN1 proteins were detected. For simplicity, species are classified as autotrophs, however, for some species of plants, alternative metabolic strategies have been identified (see Discussion).

**Figure S5.** Expanded phylogenetic tree of Alveolata and Rhizaria. Species labels are color-coded by BUSCO completeness scores to reflect assembly quality. The first heatmap displays HMMER-based detection scores for TOR complex components: SIN1, RICTOR, RAPTOR, LST8, and TOR. The second heatmap shows inferred metabolic strategies, based on literature review and phylogenetic context. Among all groups analyzed, Alveolata include the most species lacking both TOR complexes, as well as the only species retaining TORC2 but lacking TORC1. In contrast, all Rhizaria species surveyed retain both complexes, despite lower average completeness scores.

**Figure S6**. Expanded phylogenetic tree of Stramenopiles. Species labels are color-coded by BUSCO completeness scores to indicate assembly quality. The first heatmap shows HMMER-based detection scores for core TOR complex components: SIN1, RICTOR, RAPTOR, LST8, and TOR. The second heatmap depicts inferred metabolic strategies, based on literature review and phylogenetic analysis. Stramenopiles exhibit variable retention of TORC1 and TORC2 components, with multiple independent loss events of TORC2 observed within the clade.

**Figure S7.** Composite phylogenetic tree of parasitic and endosymbiotic species among eukaryotic supergroups. Phylogenetic tree includes all species classified as a parasite or endosymbiont from among the supergroups examined in this study. For formatting purposes, species labels are omitted; terminal branches are color-coded by BUSCO completeness score. A full list of included species is provided in Table S1.

**Disclaimer/Publisher's Note:** The statements, opinions and data contained in all publications are solely those of the individual author(s) and contributor(s) and not of MDPI and/or the editor(s). MDPI and/or the editor(s) disclaim responsibility for any injury to people or property resulting from any ideas, methods, instructions or products referred to in the content.
