# Supplementary figures and images for: Metabolic Adaptations Determine the Evolutionary Trajectory of TOR Signaling in Diverse Eukaryotes
Source: Biomolecules. 2025 Sep 8;15(9):1295. doi: 10.3390/biom15091295 (PMC12467227; doi:10.3390/biom15091295)

Figure S1

(A)

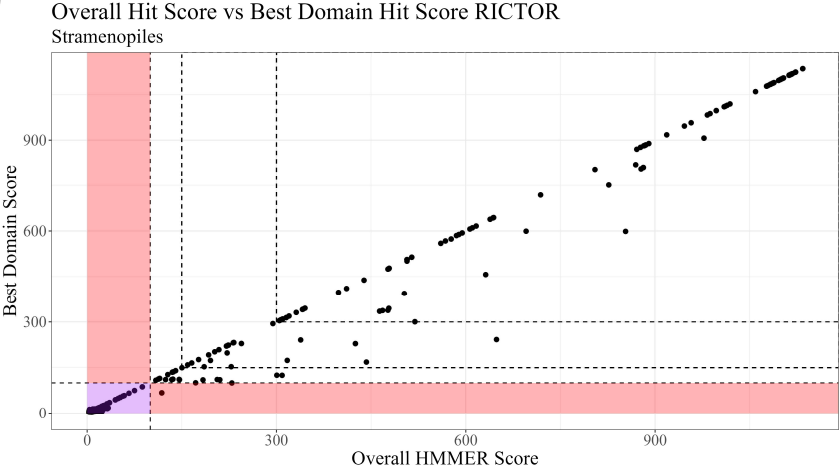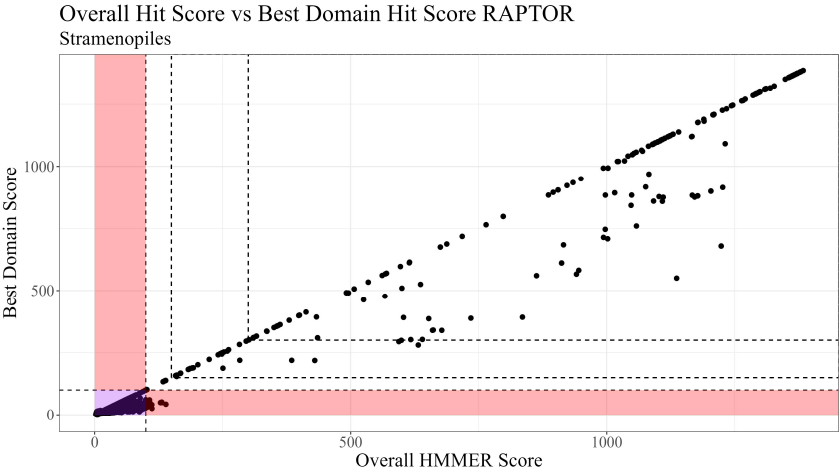

(B)

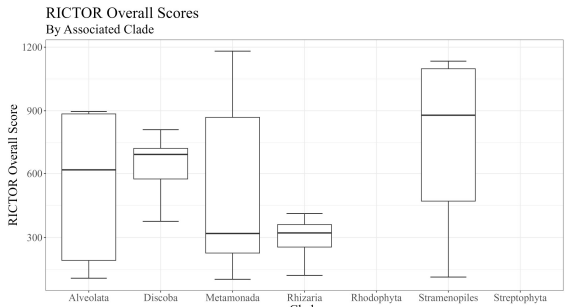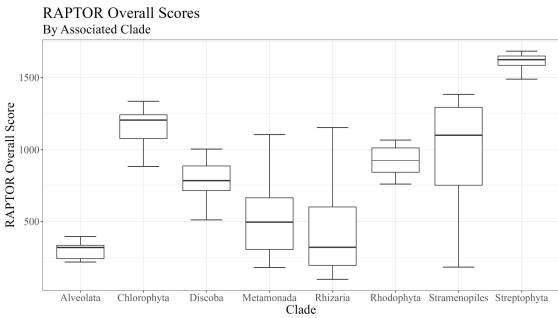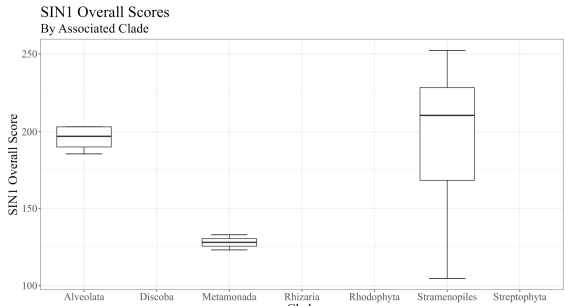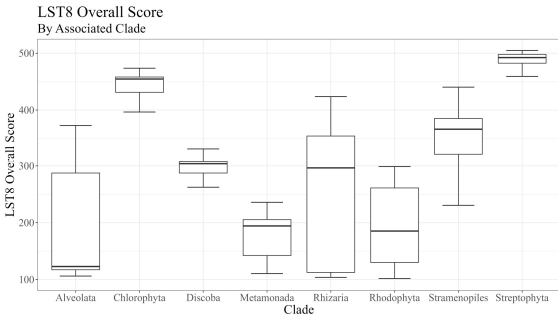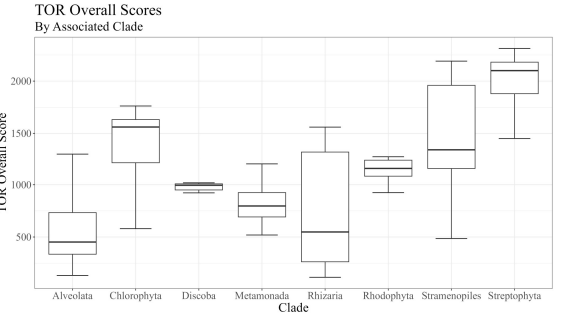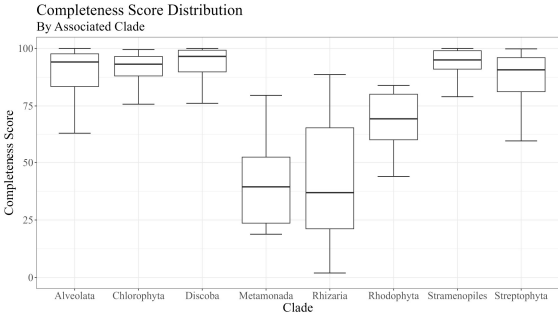



Figure S3

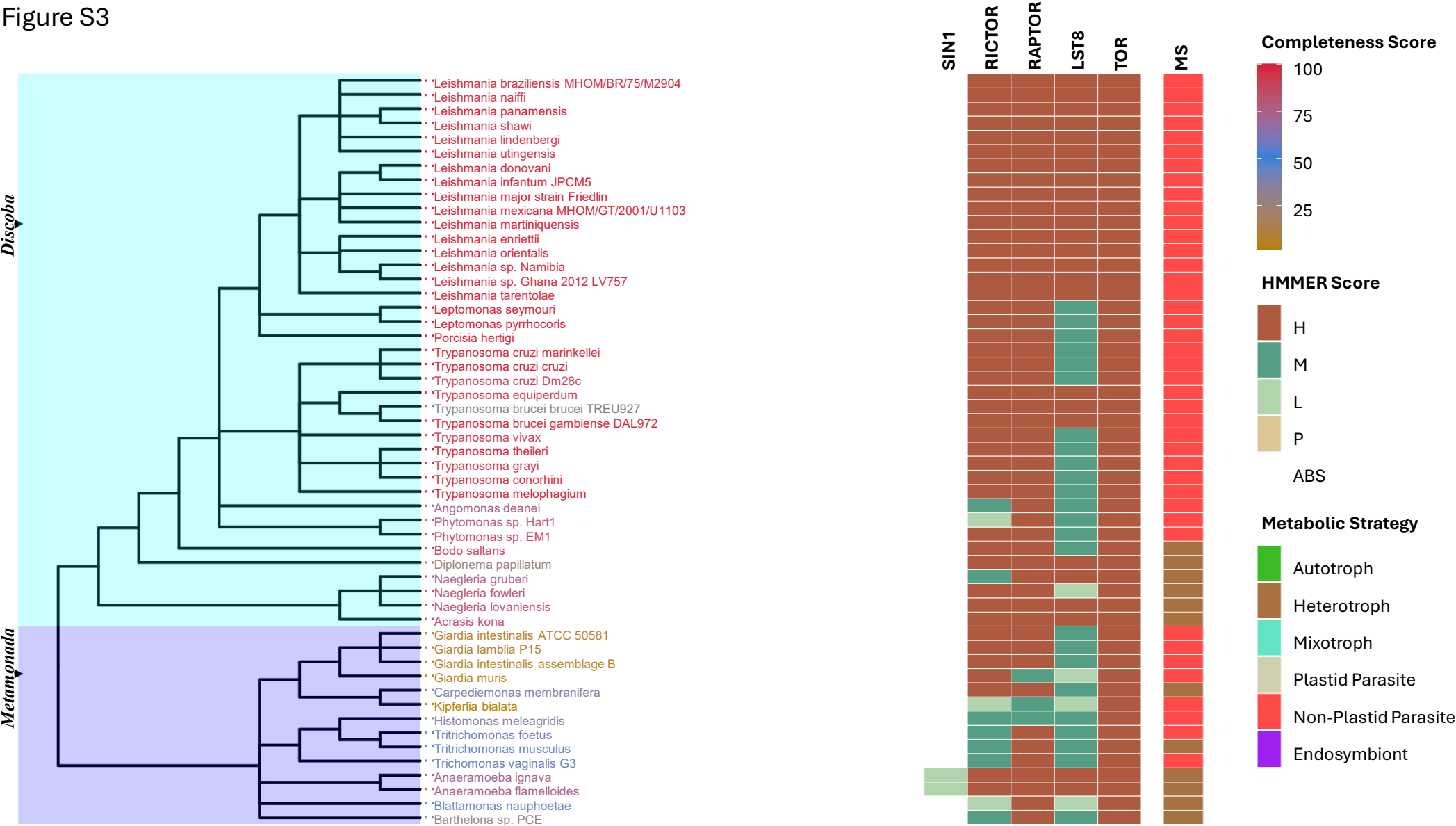

Figure S4

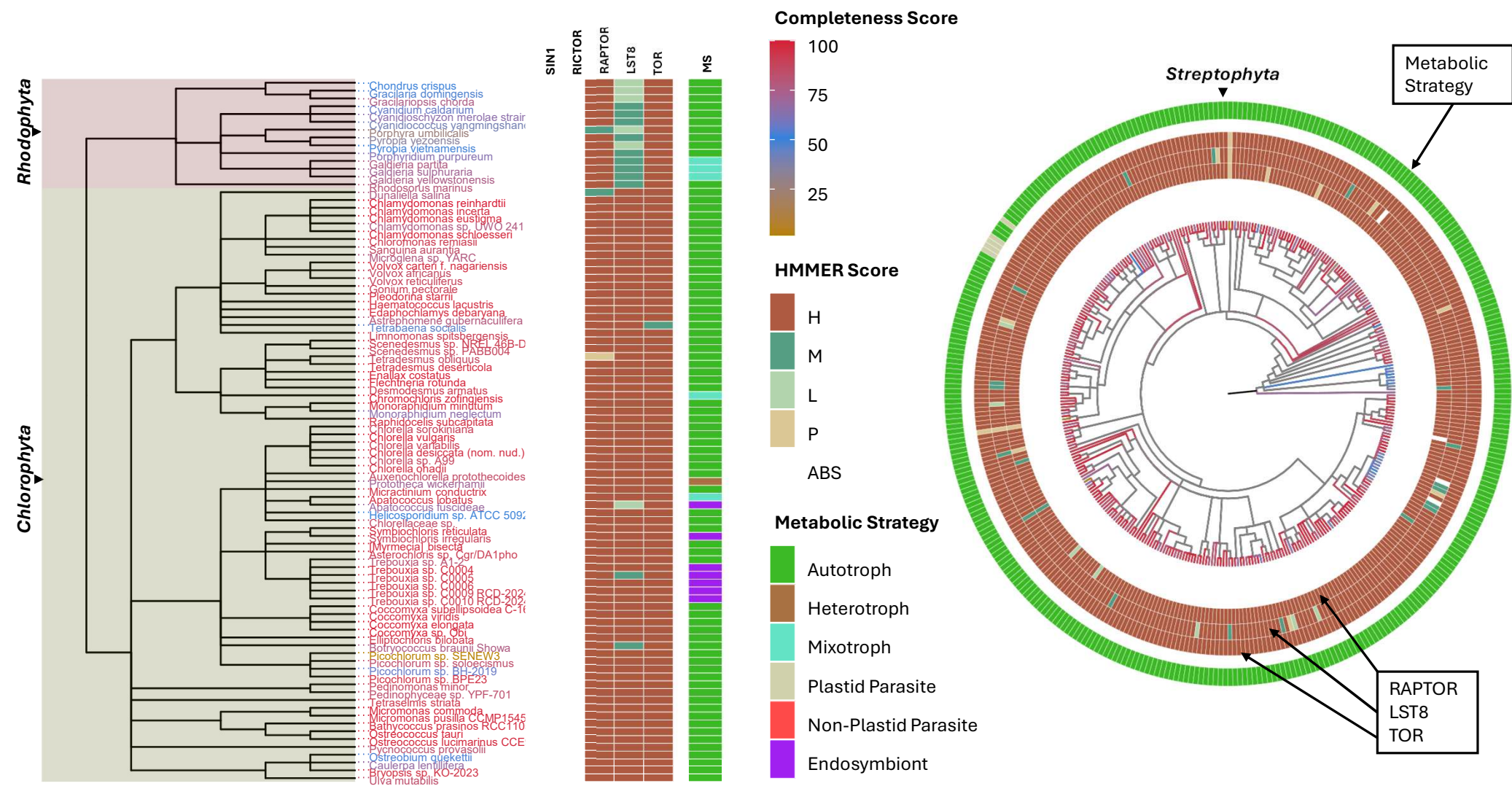

Figure S5

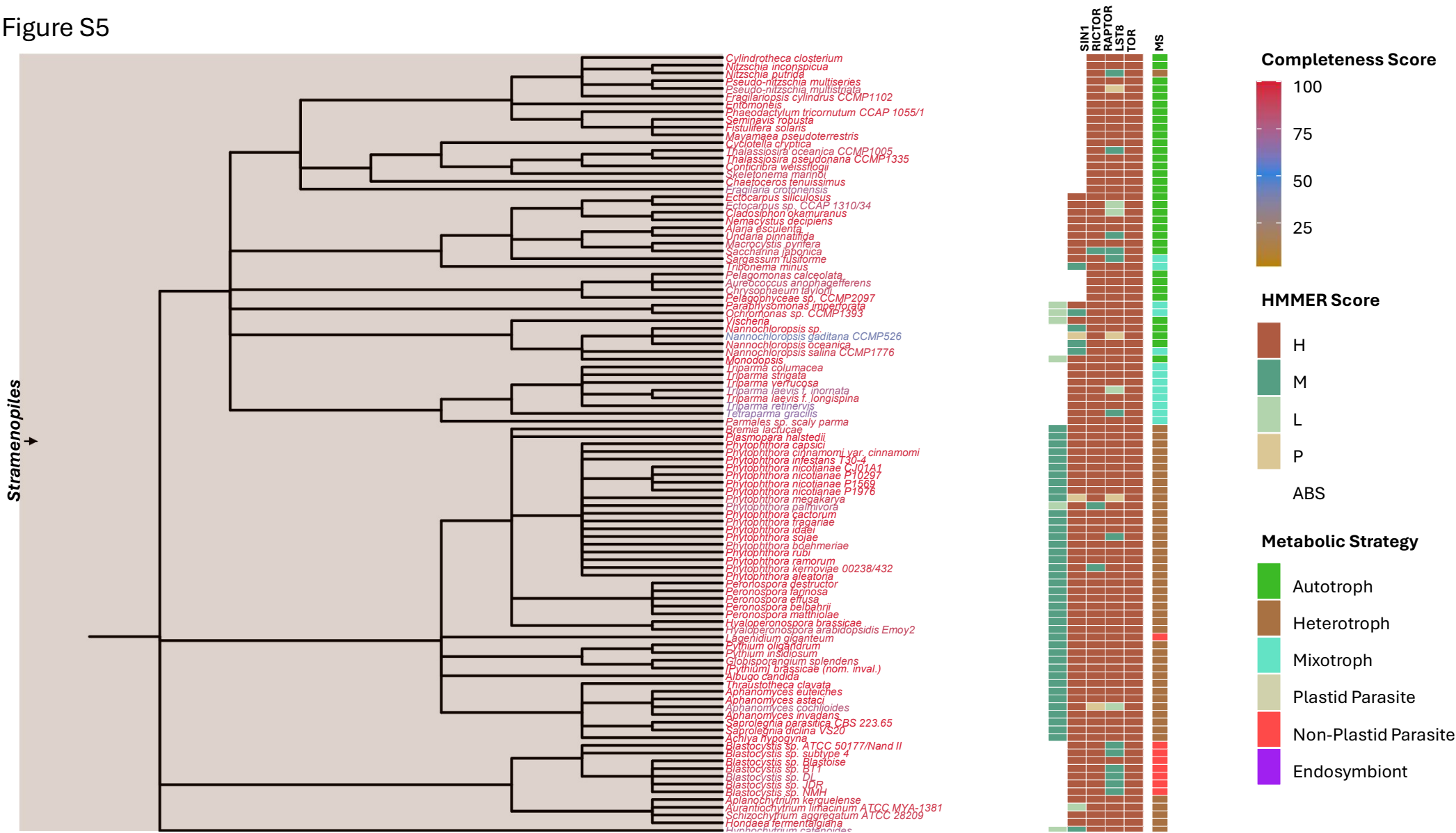

Figure S6

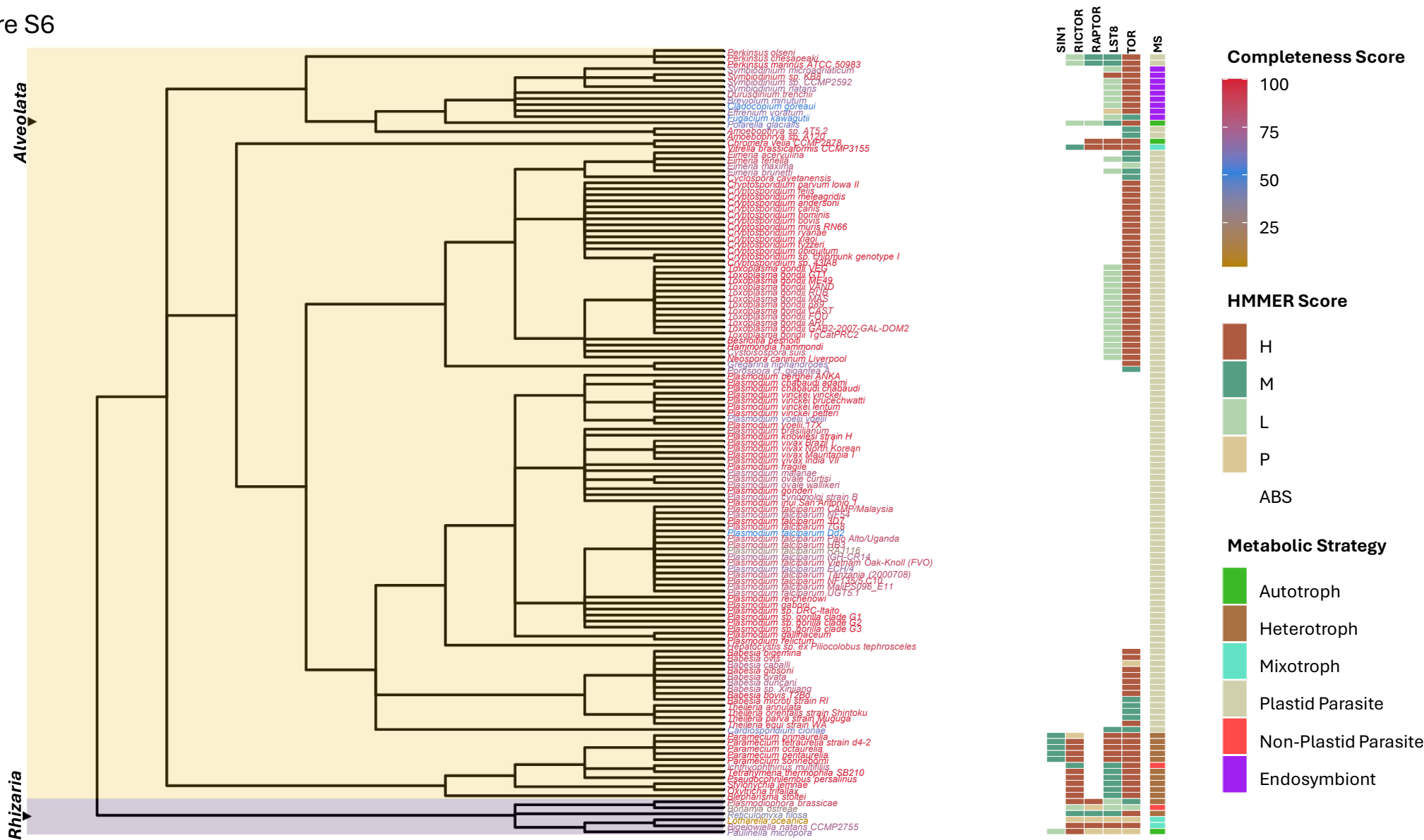

Figure S7

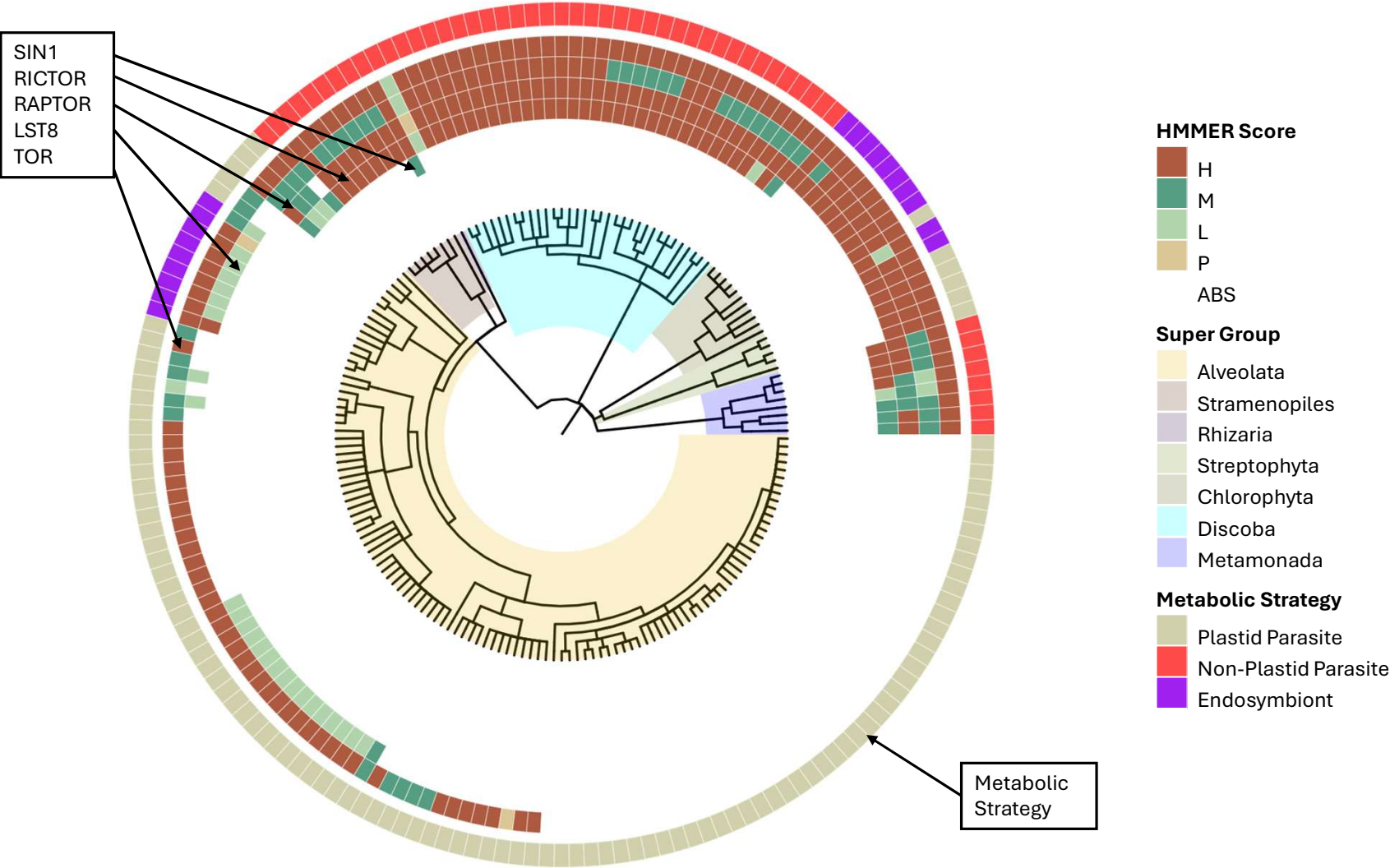

Supplement: Supplementary file 1 [file biomolecules-15-01295-s001.zip › Supplemental_Figures.pdf]
